# Supplementary material for: Distinct population and single-neuron selectivity for executive and episodic processing in human dorsal posterior cingulate
Source: eLife. 2022 Sep 28;11:e80722. doi: 10.7554/eLife.80722 (PMC9519147; doi:10.7554/eLife.80722)
Supplement: Supplementary file 1. — Demographic and electrode information is reported for each subject (1-20), including sex (male/female), age at time of experiment (years), total number of PCC electrodes, number of dPCC electrodes, number of vPCC electrodes, and number of microwire probes. [file elife-80722-supp1.docx]

**Supplementary Files**

| **Subject** | **Sex** | **Age (yrs.)** | **#Total**  **PCC Electrodes** | **#dPCC**  **Electrodes** | **#vPCC**  **Electrodes** | **#Microwire probes** |
| --- | --- | --- | --- | --- | --- | --- |
| 1 | F | 33 | 1 | 1 | - | 1 |
| 2 | M | 30 | 2 | 2 | - | 1 |
| 3 | M | 22 | 1 | 1 | - | 1 |
| 4 | M | 39 | 1 | 1 | - | - |
| 5 | F | 21 | 1 | 1 | - | 1 |
| 6 | M | 30 | 1 | 1 | - | 1 |
| 7 | M | 40 | 1 | 1 | - | 1 |
| 8 | M | 24 | 8 | 6 | 2 | 2 |
| 9 | M | 43 | 3 | 2 | 1 | 1 |
| 10 | F | 56 | 1 | 1 | - | 1 |
| 11 | M | 26 | 1 | 1 | - | 1 |
| 12 | M | 19 | 2 | - | 2 | - |
| 13 | M | 24 | 2 | 1 | 1 | 1 |
| 14 | F | 52 | 1 | 1 | - | - |
| 15 | M | 23 | 1 | 1 | - | 1 |
| 16 | F | 53 | 1 | 1 | - | 1 |
| 17 | F | 25 | 2 | 2 | - | - |
| 18 | F | 41 | 1 | 1 | - | - |
| 19 | M | 37 | 2 | 2 | - | - |
| 20 | F | 44 | 2 | 2 | - | - |
| **Total/Avg** | **12M/8F** | **34.1** | **35** | **29** | **6** | **14** |

**Supplementary file 1. Subject demographic and electrode information.**

Demographic and electrode information is reported for each subject (1-20), including sex (male/female), age at time of experiment (years), total number of PCC electrodes, number of dPCC electrodes, number of vPCC electrodes, and number of microwire probes.
